# Supplementary material for: From sequence to enzyme mechanism using multi-label machine learning
Source: BMC Bioinformatics. 2014 May 19;15:150. doi: 10.1186/1471-2105-15-150 (PMC4229970; doi:10.1186/1471-2105-15-150)
Supplement: Additional file 2 — Java code of ml2db. Additional file ml2db_code.tar.gz contains the Java source code to run the multi-label machine learning experiments and save the results to database. The code’s Javadoc is included. [file 1471-2105-15-150-S2.zip › additional file 2/ml2db/utils/doc/uk/ac/ed/inf/utils/TimeUtils.html]

TimeUtils


---


|  |  |  |
| --- | --- | --- |
| **Overview** \| **Package** \| **Class** \| **Use** \| **Tree** \| **Deprecated** \| **Index** \| **Help** | |  |
| **PREV CLASS**   **NEXT CLASS** | **FRAMES**    **NO FRAMES**     **All Classes** |  |
| SUMMARY: NESTED \| FIELD \| CONSTR \| METHOD | DETAIL: FIELD \| CONSTR \| METHOD |  |


---


## uk.ac.ed.inf.utils Class TimeUtils

```
java.lang.Object
  uk.ac.ed.inf.utils.TimeUtils
```

---

``` public class TimeUtils extends java.lang.Object ```

Utilities to manipulate time objects, create timestamps etc.

**Version:**
:   4 Jan 2008

**Author:**
:   Luna De Ferrari luna.deferrari-at-ed.ac.uk

---

| **Field Summary** | |
| --- | --- |
| `static java.lang.String` | `DAY` |
| `static java.lang.String` | `HOUR` |
| `static java.lang.String` | `MILLISECONDS` |
| `static java.lang.String` | `MINUTE` |
| `static java.lang.String` | `MONTH` |
| `static java.lang.String` | `SECOND` |
| `static java.lang.String` | `YEAR` |


**Constructor Summary**

`TimeUtils()`


| **Method Summary** | |
| --- | --- |
| `static java.lang.String` | `from1DigitTo2DigitsDate(java.lang.String date1digit)`             Returns a day or month number in 2 digits format. |
| `static java.lang.String` | `fromDMYYYtoYYYYMMDDdate(java.lang.String DMYYYYdate, java.lang.String inputDateSeparator, java.lang.String outputDateSeparator)`             Converts from D/M/YYYY date (6/19/2006) to YYYY-MM-DD date (2006-06-19) |
| `static java.lang.String` | `getDateForFileName()`             getFileTimeStamp returns a date stamp that can be attached to file names |
| `static java.lang.String` | `getDateHourStampForFileName()`             Date-time stamp (up to hours) that can be attached to file names |
| `static java.lang.String` | `getDateStamp()`             Returns a date stamp in the format: 20070117 yyyymmdd |
| `static java.util.HashMap<java.lang.String,java.lang.String>` | `getDateStrings()`             getDateStrings() |
| `static java.lang.String` | `getDateTimeMillisecondsStamp()`             Returns a date-time-milliseconds stamp in the format: 20070117\_111847\_123 yyyymmdd\_hhmmss\_lll |
| `static java.lang.String` | `getDateTimeNanoSecondsStamp()`             Returns a date-time-milliseconds-nanoseconds stamp in the format: 20070117\_111847\_123\_3000 yyyymmdd\_hhmmss\_lll\_nnnn (the n digits are not the nanoseconds! just a progressive stamp) |
| `static java.lang.String` | `getDateTimeStamp()`             Returns a date-time stamp in the format: 20070117\_111847 yyyymmdd\_hhmmss |
| `static java.lang.String` | `getElapsedTime(long startTime, long endTime)`             Returns a string with the elapsed time in a hour:min:sec.msec format |
| `static java.lang.String` | `getHourOnlyStamp()`             Returns a time stamp in the format (for 11 am, 18 min, 47 sec): 11 hh |
| `static java.lang.String` | `getHumanReadableTimeStamp()`             Returns a human readable date-time stamp |
| `static java.lang.String` | `getMilliseconds()`             Returns a milliseconds time stamp |
| `static java.lang.String` | `getMillisecTimeStampForFileName()`             Date-time stamp (up to milliseconds) that can be attached to file names |
| `static java.lang.String` | `getNanoSecondsStamp()`             Returns a nanoseconds stamp (it's a progressive stamp actually, not the actual nanoseconds in desktop-clock style!) |
| `static java.lang.String` | `getNextNanoSecondsStamp(java.lang.String nanoTimeStamp)`             Returns a nanoseconds stamp + 1 (it's a progressive stamp actually, not the actual nanoseconds in desktop-clock style!) |
| `static java.sql.Timestamp` | `getSqlTimestamp()`             Gets a sql.Timestamp object of the current time (precise to the second: nanoseconds are set to zero) |
| `static java.lang.String` | `getSqlTimestampString()` |
| `static java.lang.String` | `getTimeOnlyStamp()`             Returns a time stamp in the format (for 11 am, 18 min, 47 sec): 111847 hhmmss |
| `static java.lang.String` | `getTimePassed(java.util.Calendar startTime)` |
| `static java.lang.String` | `getTimeStampForFileName()`             Date-time stamp (up to seconds) that can be attached to file names |
| `static void` | `printTimestampedComment(java.lang.String comment)`             Writes to System.out a timestamp + tab + the comment. |
| `static void` | `printTimestampedError(java.lang.String comment)` |
| `static java.lang.String` | `timestampedComment(java.lang.String comment)` |

**Methods inherited from class java.lang.Object**

`equals, getClass, hashCode, notify, notifyAll, toString, wait, wait, wait`

**Field Detail**

### DAY

```
public static final java.lang.String DAY
```

**See Also:**: Constant Field Values

---


### HOUR

```
public static final java.lang.String HOUR
```

**See Also:**: Constant Field Values

---


### MILLISECONDS

```
public static final java.lang.String MILLISECONDS
```

**See Also:**: Constant Field Values

---


### MINUTE

```
public static final java.lang.String MINUTE
```

**See Also:**: Constant Field Values

---


### MONTH

```
public static final java.lang.String MONTH
```

**See Also:**: Constant Field Values

---


### SECOND

```
public static final java.lang.String SECOND
```

**See Also:**: Constant Field Values

---


### YEAR

```
public static final java.lang.String YEAR
```

**See Also:**: Constant Field Values


**Constructor Detail**

### TimeUtils

```
public TimeUtils()
```


**Method Detail**

### from1DigitTo2DigitsDate

```
public static java.lang.String from1DigitTo2DigitsDate(java.lang.String date1digit)
```

:   Returns a day or month number in 2 digits format. Example: month 5
    becomes month 05.

    :   **Parameters:**: `date1digit` - **Returns:**: a day or month number in 2 digits format

---


### fromDMYYYtoYYYYMMDDdate

```
public static java.lang.String fromDMYYYtoYYYYMMDDdate(java.lang.String DMYYYYdate,
                                                       java.lang.String inputDateSeparator,
                                                       java.lang.String outputDateSeparator)
```

:   Converts from D/M/YYYY date (6/19/2006) to YYYY-MM-DD date (2006-06-19)

    :   **Parameters:**: `DMYYYYdate` - a D M YYYY date (example: 6/19/2006): `inputDateSeparator` - (example: / ): `outputDateSeparator` - (example: - ) **Returns:**: a YYYY MM DD date (2006-06-19)

---


### getDateForFileName

```
public static java.lang.String getDateForFileName()
```

:   getFileTimeStamp returns a date stamp that can be attached to file names

    :   **Returns:**: a string with date in the format: \_20070117 \_yyyymmdd)

---


### getDateStamp

```
public static java.lang.String getDateStamp()
```

:   Returns a date stamp in the format: 20070117 yyyymmdd

    :   **Returns:**: a string with date in the format: 20070117 yyyymmdd)

---


### getDateStrings

```
public static java.util.HashMap<java.lang.String,java.lang.String> getDateStrings()
```

:   getDateStrings()

    :   **Returns:**: a hashmap containing the date-time elements as strings

---


### getDateTimeMillisecondsStamp

```
public static java.lang.String getDateTimeMillisecondsStamp()
```

:   Returns a date-time-milliseconds stamp in the format: 20070117\_111847\_123
    yyyymmdd\_hhmmss\_lll

    :   **Returns:**: a date-time-milliseconds stamp in the format: 20070117\_111847\_123 yyyymmdd\_hhmmss\_lll

---


### getDateTimeNanoSecondsStamp

```
public static java.lang.String getDateTimeNanoSecondsStamp()
```

:   Returns a date-time-milliseconds-nanoseconds stamp in the format:
    20070117\_111847\_123\_3000 yyyymmdd\_hhmmss\_lll\_nnnn (the n digits are not
    the nanoseconds! just a progressive stamp)

    :   **Returns:**: a date-time-milliseconds stamp in the format: 20070117\_111847\_123 yyyymmdd\_hhmmss\_lll\_n

---


### getDateTimeStamp

```
public static java.lang.String getDateTimeStamp()
```

:   Returns a date-time stamp in the format: 20070117\_111847 yyyymmdd\_hhmmss

    :   **Returns:**: a string with date in the format: 20070117\_111847 yyyymmdd\_hhmmss

---


### getElapsedTime

```
public static java.lang.String getElapsedTime(long startTime,
                                              long endTime)
```

:   Returns a string with the elapsed time in a hour:min:sec.msec format

    :   **Parameters:**: `startTime` - a time in milliseconds (System.currentTimeMillis()): `endTime` - a time in milliseconds (System.currentTimeMillis()) **Returns:**

---


### getHumanReadableTimeStamp

```
public static java.lang.String getHumanReadableTimeStamp()
```

:   Returns a human readable date-time stamp

    :   **Returns:**: a string with date and time in the format: YYYY-MM-DD hh:mm:ss

---


### getMilliseconds

```
public static java.lang.String getMilliseconds()
```

:   Returns a milliseconds time stamp

    :   **Returns:**: a milliseconds time stamp

---


### getMillisecTimeStampForFileName

```
public static java.lang.String getMillisecTimeStampForFileName()
```

:   Date-time stamp (up to milliseconds) that can be attached to file names

    :   **Returns:**: a string with date and time in the format: \_20070117\_111847 \_yyyymmdd\_hhmmss)

---


### getNanoSecondsStamp

```
public static java.lang.String getNanoSecondsStamp()
```

:   Returns a nanoseconds stamp (it's a progressive stamp actually, not the
    actual nanoseconds in desktop-clock style!)

    :   **Returns:**: a nanoseconds stamp

---


### getNextNanoSecondsStamp

```
public static java.lang.String getNextNanoSecondsStamp(java.lang.String nanoTimeStamp)
```

:   Returns a nanoseconds stamp + 1 (it's a progressive stamp actually, not
    the actual nanoseconds in desktop-clock style!)

    :   **Returns:**: a nanoseconds stamp

---


### getSqlTimestamp

```
public static java.sql.Timestamp getSqlTimestamp()
```

:   Gets a sql.Timestamp object of the current time (precise to the second:
    nanoseconds are set to zero)

    :   **Returns:**: a sql.Timestamp object of the current time

---


### getSqlTimestampString

```
public static java.lang.String getSqlTimestampString()
```

---


### getTimeOnlyStamp

```
public static java.lang.String getTimeOnlyStamp()
```

:   Returns a time stamp in the format (for 11 am, 18 min, 47 sec): 111847
    hhmmss

    :   **Returns:**: a string with time in the format (for 11 am, 18 min, 47 sec): 111847 hhmmss

---


### getHourOnlyStamp

```
public static java.lang.String getHourOnlyStamp()
```

:   Returns a time stamp in the format (for 11 am, 18 min, 47 sec): 11 hh

    :   **Returns:**: a string with time in the format (for 11 am, 18 min, 47 sec): 11 hh

---


### getTimePassed

```
public static java.lang.String getTimePassed(java.util.Calendar startTime)
```

---


### getTimeStampForFileName

```
public static java.lang.String getTimeStampForFileName()
```

:   Date-time stamp (up to seconds) that can be attached to file names

    :   **Returns:**: a string with date and time in the format: \_20070117\_111847 \_yyyymmdd\_hhmmss)

---


### getDateHourStampForFileName

```
public static java.lang.String getDateHourStampForFileName()
```

:   Date-time stamp (up to hours) that can be attached to file names

    :   **Returns:**: a string with date and hour in the format: \_20070117\_11 \_yyyymmdd\_hh)

---


### printTimestampedComment

```
public static void printTimestampedComment(java.lang.String comment)
```

:   Writes to System.out a timestamp + tab + the comment.

    :   **Parameters:**: `comment` -

---


### printTimestampedError

```
public static void printTimestampedError(java.lang.String comment)
```

---


### timestampedComment

```
public static java.lang.String timestampedComment(java.lang.String comment)
```


---


|  |  |  |
| --- | --- | --- |
| **Overview** \| **Package** \| **Class** \| **Use** \| **Tree** \| **Deprecated** \| **Index** \| **Help** | |  |
| **PREV CLASS**   **NEXT CLASS** | **FRAMES**    **NO FRAMES**     **All Classes** |  |
| SUMMARY: NESTED \| FIELD \| CONSTR \| METHOD | DETAIL: FIELD \| CONSTR \| METHOD |  |


---
